# DETECTION OF FORWARD PROPULSION USING A SINGLE ACCELEROMETER DURING WALKING IN OLDER POPULATION

**DOI:** 10.1093/geroni/igz038.1211

**Published:** 2019-11-08

**Authors:** Mohsen Zahiri, Bijan Najafi, Hung Nguyen, Gu Eon Kang

**Affiliations:** Baylor College of Medicine, Houston, Texas, United States

## Abstract

In the geriatric population, diminished ankle joint moment and weak plantar flexor can contribute to inadequate forward propulsion and negatively impact gait performance, which can lead to poor energetic efficiency. Detection of propulsion phase can help identify gait normality and guide rehabilitation therapy to improve functional performance. Current methods have limited application in daily life and unsuitable for continuous monitoring. In this study, we aim to develop algorithms based on a single sensor attached to the shin to accurately detect propulsion phase. Six elderly (age: 73 years, BMI: 30.4) were recruited. Participants walked at their normal pace while wearing a plantar pressure system and an accelerometer on the shin. The pressure data was used to define the beginning of the propulsion phase when the pressure switched from the heel to the forefoot. A wavelet algorithm was developed to automatically detect the start and end points of propulsion phase using an accelerometer. The Bland-Altman method was used to evaluate the agreement between these methods. Pearson’s Coefficient was used to quantify the correlation. Based on the Bland-Altman analysis, A high agreement was obtained between the proposed method using accelerometer and pressure sensor (bias =9 ms, precision = 30 ms). Both algorithms are significantly correlated (r = 0.85, p<0.05). This study presents an innovative algorithm to automatically detect the propulsion phase for older adults during walking. Using wearable could facilitate the capture of propulsion phase during living activity, which might provide more insights into the mechanism of walking during rehabilitation therapy.

